# Relationship between abnormal osteoblasts and cellular immunity in multiple myeloma

**DOI:** 10.1186/1475-2867-14-62

**Published:** 2014-08-14

**Authors:** Rong Fu, Shan Gao, Fengping Peng, Jing Li, Hui Liu, Huaquan Wang, Linmin Xing, Zonghong Shao

**Affiliations:** 1 Department of Hematology, General Hospital, Tianjin Medical University, 154 Anshan Street, Heping District, Tianjin 300052, China

**Keywords:** Multiple myeloma, Osteoblast, Cellular immunity, Bortezomib

## Abstract

Bone destruction and abnormal immunity always occur in patients with multiple myeloma (MM), which manifested by impaired osteoblasts and immune system. In this study, we investigated the quantity and function of osteoblasts by co-culture, the status of cellular immunity by flow cytometry, and the relationship between them in MM patients. The results showed that the numbers and function of osteoblasts in MM patients were lower than those in normal controls. Bortezomib could increase the numbers, calcium depositions and the expression of Bone morphogenetic protein–2 (BMP-2) mRNA of osteoblasts from MM patients *in vitro*. The status of cellular immunity in MM patients was abnormal, including decreased ratio of CD4^+^/CD8^+^, DC1/DC2 and Th1/Th2, and increased ratio of regulatory T cells. The ratio of CD4^+^/CD8^+^(r = 0.685) and CD4^+^CD25^+^/CD3^+^T(r = 0.568) were positively correlated with the quantity of osteoblasts (both P < 0.05). The serum interleukin-7(IL-7) level of MM patients was higher than that of normal controls (2.07 ± 0.71 vs. 1.62 ± 0.15 ng/L, P < 0.05), and was negatively correlated with the quantity of osteoblasts (r = -0.682, P < 0.01). Our data indicated that the proliferation and osteogenic potential of osteoblasts in MM patients were decreased which could be recovered by bortezomib *in vitro*. The down-regulation of cellular immunity was correlated with the quantity of osteoblasts.

## Introduction

Multiple myeloma (MM) is a type of plasma malignant tumor. Myeloma bone disease (MBD) is the most common complication of MM. Over 80% MM patients suffered from bone destruction associated with diffuse osteopenia, focal lytic lesions, pathologic fractures, hypercalcemia, and bone pain. The past studies showed that myeloma cells, bone marrow (BM) matrix cells and some cytokines secreted by osteoblasts or osteoclasts could increase osteoclasts activity and inhibit osteoblast in the microvironment of BM. MM patients with osteolytic bone lesions usually have decreased numbers of osteoblasts and impaired bone formation accompanied by increased activation of osteoclasts [1]. Histomorphometric studies have demonstrated that MM patients with MBD had uncoupled or severe imbalanced bone remodeling with increased bone resorption and decreased or absent bone formation [2]. Furthermore, recent studies showed that MM patients had polydeficiency in immune system which could influence the progression of the disease, including the quantity and function disturbance of B lymphocytes, T lymphocytes, natural killer cells (NK cells), dendritic cells (DC) and regulatory T cells (Tregs) [3]. But the relationship between MBD and abnormal immunity is still unclear.

The traditional treatments of MBD include calcium, 1,25-(OH)_2_D_3_, calcitionin and diphosphonate to reduce bone destruction. In recent, bortezomib, as a proteasome inhibitor, has been proved that could stimulate osteoblast differentiation and promot bone formation besides destroy myeloma cells. In this study, we investigated the number and function of obsteoblasts, the factors which could regulate the proliferation and osteogenic potential of obsteoblasts (including bortezomib, serum of MM patients), and the relationship between abnormal cellular immunity (ACI) and MBD in MM patients.

## Materials and methods

### Patients

Twenty patients (12 males and 8 females, median age 57, age range 50–71) newly-diagnosed with MM at the Hematology Department of General Hospital, Tianjin Medical University, Tianjin, China, from August 2009 to August 2012 were enrolled in this study. Ten healthy donors (7 males and 3 females, median age 44, age range 18–68) were selected as controls. This study was approved by Ethical Committee of Tianjin Medical University General Hospital.

### Cell culture

Mononuclear cells isolated from BM of MM patients were cultured as 3 × 10^5^ cells/cm^2^ in 6-well culture plates with DMEM (Gibco, US), 10% fetal calf serum(Gibco, US), 1 × 10^-7^ mmol/L dexamethasone, 0.01 mol/L β- glycerophosphate, 0.05 g/L vitamin C, 100 U/L penicillin and phytomycin, at 37°C, 5% CO_2_. The cultures were replaced by fresh medium 2 times a week. Until confluence, osteoblasts were subcultured as 1 × 10^4^ cells/cm^2^ in 6-well culture plates under the same conditions. After twice subcultures, osteoblasts from MM patients or normal subjects were divided into 3 groups respectively: pure osteoblasts culture, osteoblasts co-cultured with 0.5 ug/mL bortezomib (Johnson&Johnson, US), osteoblasts co-cultured with 50 ug/mL MM patient’s serum.

The cultures were observed by inverted microscope and token pictures every day. The cell growth curve was drawn to calculate the cell doubling time. After twice subcultures, osteoblasts were identified by flow cytometry (FCM). Intracellular alkaline phosphatase (ALP) activity (qualitatively and quantitatively, by fast violet staining). I-type collagen (by immunohistochemistry staining), mineralization (by von Kossa staining) were also measured.

### Flow cytometry (FCM)

Osteoblasts were acquired and washed with PBS three times. All samples were divided into control and test tubes. Antibodies against mouse IgG1-FITC, IgG1-PE and IgG1-PerCP (BD Biosciences, US) were stained as a negative control. Antibodies against CD45-PerCP, CD138-FITC, and CD34-PE (BD Biosciences, US) were stained to identify the purity of osteoblasts. Peripheral blood samples were collected in EDTA- anticoagulant tubes. The number of immune subsets were measured by FCM using anti-CD4-FITC, anti-CD8-FE and anti-CD3-PerCP (to identify the subtypes of T lymphocytes); anti-CD4-PE, anti-CD25-FITC and anti-CD127-APC (regulatory T cells); anti-Lin-FITC, anti-HLA-DR-PerCP, anti-CD11c-PE and anti-CD123-APC (subtypes of dendritic cells, DC); anti-CD3-APC, anti-CD8-PerCP, anti-IFN-γ-FITC and anti-IL-4-PE (T helper cells, Th) (BD Biosciences, US). After incubation in the dark at 4°C for 30 min, the cells were incubated with 2 ml erythrocyte lytic solution (BD Biosciences) at room temperature for 10 min. The cells were then washed two times with PBS. At least 10,000-30,000 cells were acquired and analyzed on FACSCalibur flow cytometer (BD Biosciences).

### Serum interleukin-7(IL-7) analyzed by Enzyme-linked immunosorbent assay (ELISA)

The serum of MM patients and healthy donors were separated from fresh blood samples (2 ml). The levels of IL-7 was measured by human ELISA kit (R&D Systems, US).

### Bone morphogenetic protein–2 (BMP-2) mRNA expressions of osteoblasts analyzed by Real-time transcriptase polymerase chain reaction (RT-PCR)

Total RNA was extracted from 1 × 10^6^ of osteoblasts by TRIzol reagent (Invitrogen, US), and reverse transcripted to cDNA by iScript cDNA Synthesis kit (Bio-Rad, Hercules). The primer were forward 5′- GTCCTGAGCGAGTTCGAGTT-3′, reverse 5′- TGAAGCTCTGCTGAG GTGAT -3′. The length of amplification production was 308 bp. PCR amplification conditions were: 94°C for 30 seconds, 59°C for 30 seconds and 72 C for 30 seconds for 35 cycles.

### Statistical analysis

All statistical analyses were performed using SPSS13.0 and GraphPad Prism 5 software. Data were presented as mean ± SD. The paired *t* test was used to compare two groups of paired data. The pearson correlation test was used to correlation analysis. P value of <0.05 was considered as statistically significant.

## Results

### The numbers and activity of osteoblasts from MM patients were lower than those from normal controls

**Figure 1:**
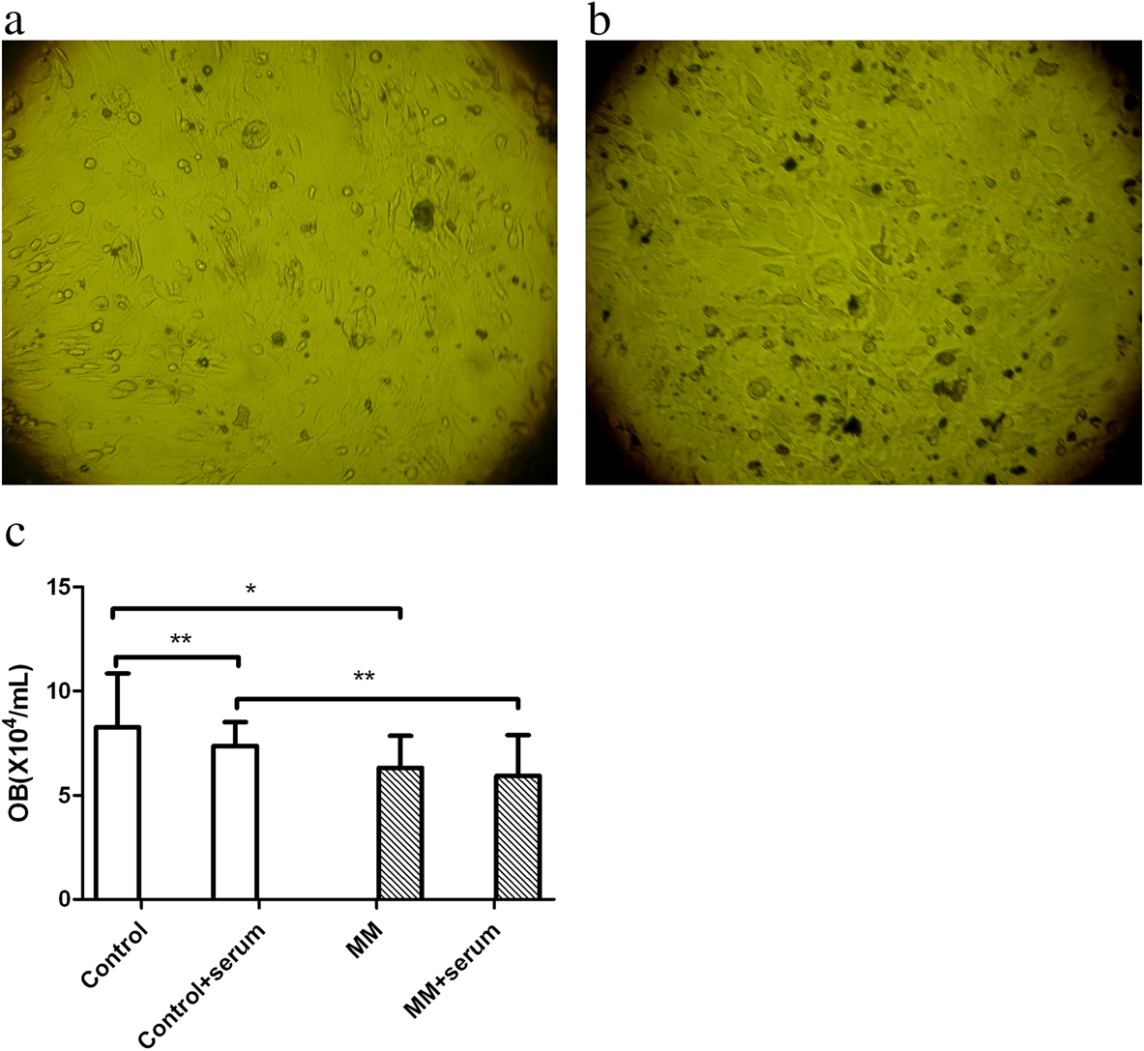
**Osteoblasts. (a,b)** The quantity of osteoblasts from MM patients was less than that of normal controls under the inverted microscope. **(c)** The quantity of osteoblasts from MM patients was less than that of normal controls (*P <0.01). After co-cultured with MM patient serum, the quantity of osteoblasts from MM patients was less than that of normal controls (**P < 0.05). And the quantity of normal osteoblasts co-cultured with MM patient serum was less than that of normal osteoblasts (**P < 0.05).

**Figure 2:**
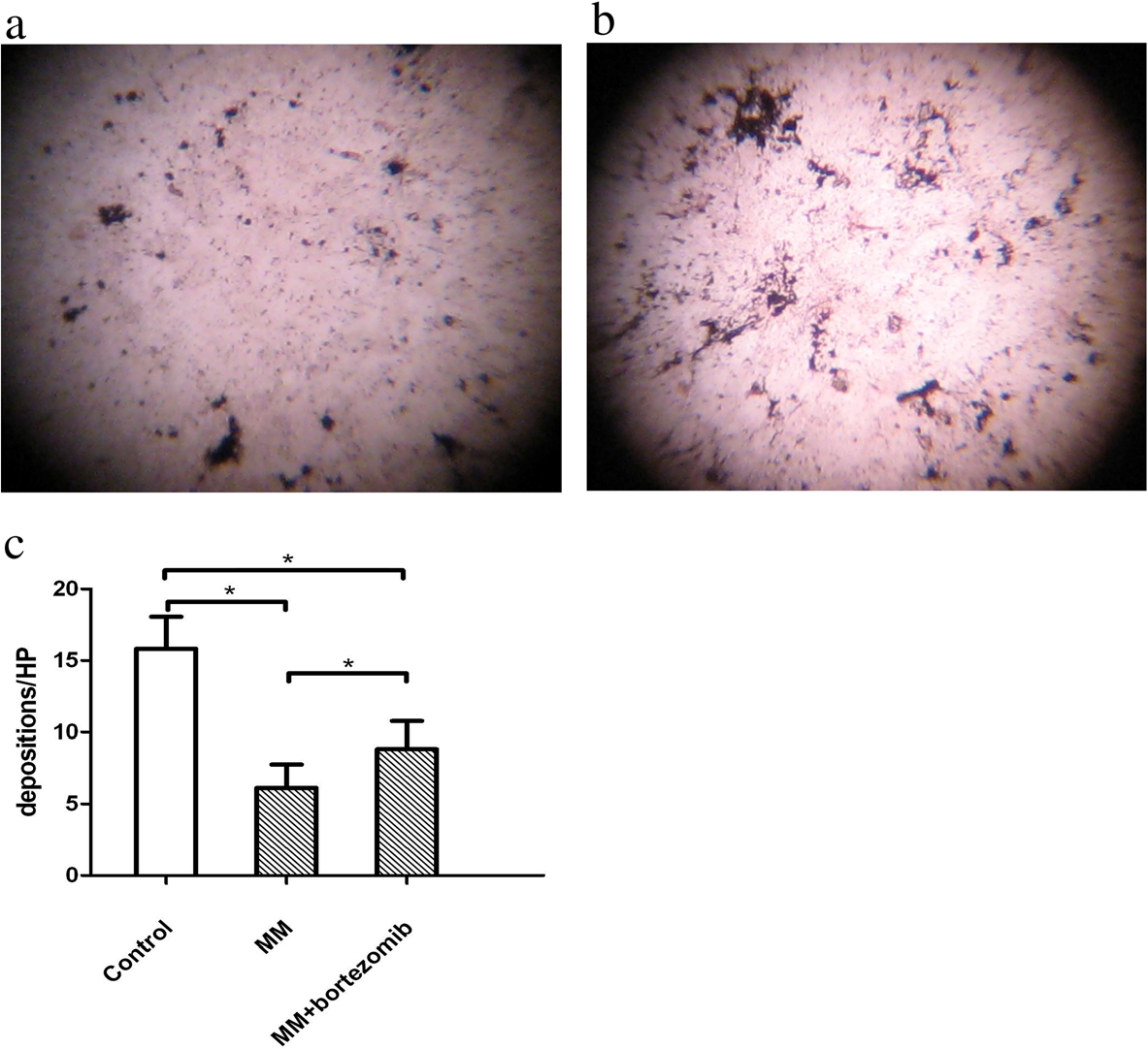
**Von Kossa staining. (a,b)** The depositions of MM patients were less than those of normal controls tested by Von Kossa staining. **(c)** The depositions of MM patients was less than those of normal controls (*P < 0.01). The calcium depositions in MM osteoblasts with bortezomib were more than those without bortezomib (*P < 0.01), less than those in normal controls (*P < 0.01).

Osteoblast’s phenotype and growth characteristics were showed in Figure 1. The osteoblasts were characterized as big, fusiform and growing parallel or spiral. The cells adhered in 24 hours and the cell doubling time was about 38 hours. After 2 weeks, the cells concentrated and formed nodes. The osteoblasts (CD45-CD138-CD34-) were identified by FCM. That showed there were neither MM cells nor blast cells in the culture which could affect the osteoblasts directly. And ALP activity staining, I-type collagen antibody staining and mineralization by von Kossa staining were positive which confirmed the cultured cells were osteoblasts.As results, the growth of osteoblast from normal controls was more prosperity than that of MM patients (Figure 1a,b). The quantity of osteoblasts was detected at the 6th day after co-culture. The quantity of osteoblasts from MM patients (6.33 ± 1.51) was less than that of normal controls (8.25 ± 2.59, P <0.01). The quantity of osteoblasts from MM patients (5.94 ± 1.64) was less than that of normal controls co-cultured with MM patient serum (7.38 ± 1.13, P < 0.05). And the quantity of normal osteoblasts co-cultured with MM patient serum was less than that of normal osteoblasts (P < 0.05) (Figure 1c).We detected calcium depositions in all cell culture systems by Von Kossa staining after 4 weeks culture. The depositions of MM patients (6.12 ± 1.63) was less than those of normal controls (15.83 ± 2.23, P < 0.01) (Figure 2a-c).

### The MM patients had abnormal immune function

**Figure 3:**
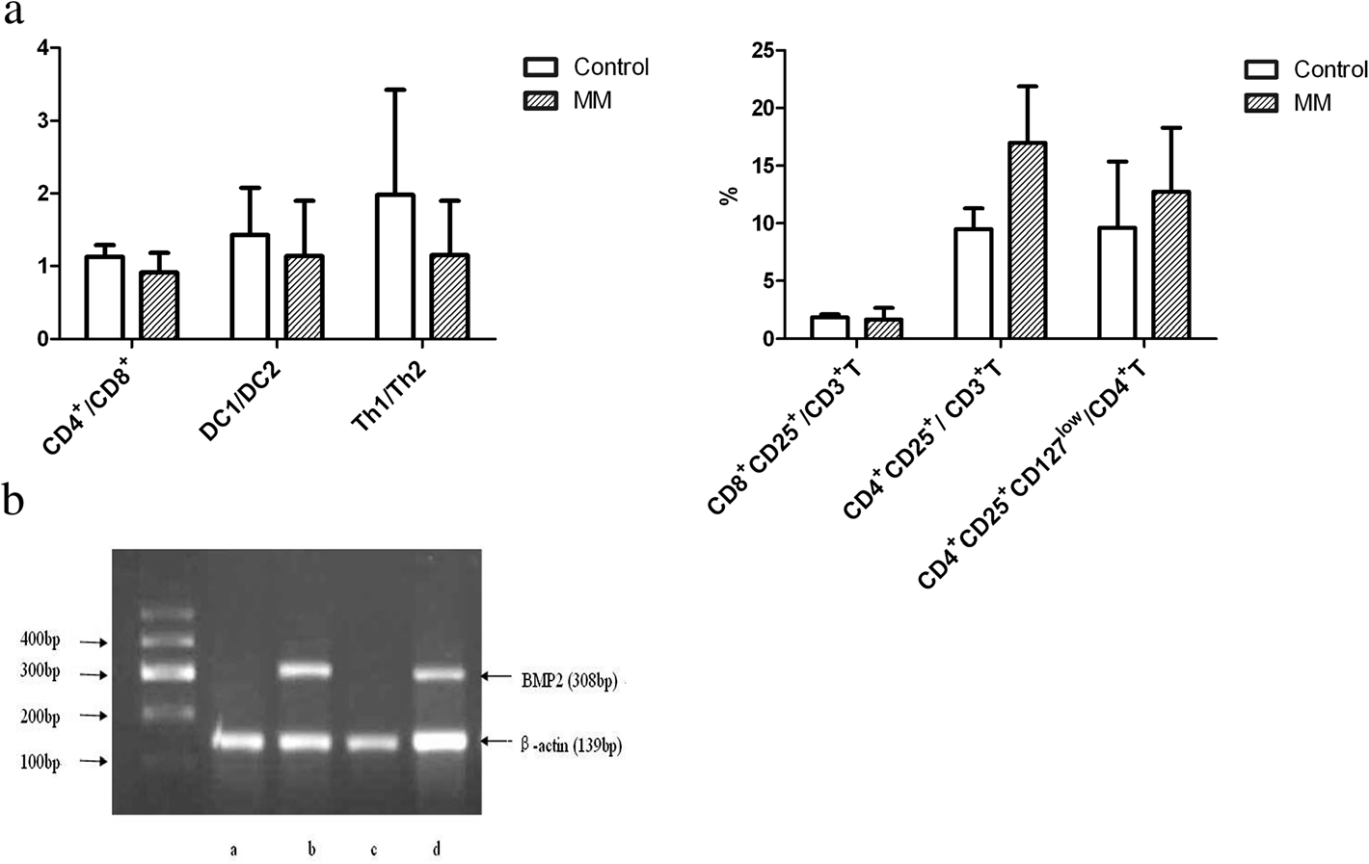
**Cellular immunity.** In MM patients, **(a)** the ratio of CD4^+^/CD8^+^, DC1/DC2 and the Th1/Th2 were significantly decreased (all P < 0.05); the ratio of CD8^+^CD25^+^/CD3^+^T were reduced, meanwhile that of CD4^+^CD25^+^/CD3^+^T and CD4^+^CD25^+^CD127^low^/CD4^+^T were significantly increased (all P < 0.05). **(b)** The serum IL-7 level of MM patients was higher than that of normal controls (*P < 0.05).

The ratio of CD4^+^/CD8^+^ in MM patients (0.91 ± 0.27) was lower than that of normal controls (1.13 ± 0.16, P < 0.05). The ratio of DC1/DC2 (1.14 ± 0.76) and Th1/Th2 (1.15 ± 0.75) in MM patients was lower than those of normal controls (1.43 ± 0.65, 1.98 ± 1.44, respectively, both P < 0.05). In peripheral blood from MM patients, the ratio of CD8^+^CD25^+^/CD3^+^T (1.13 ± 0.16%) was lower, meanwhile the ratio of CD4^+^CD25^+^/CD3^+^T (17.01 ± 4.85%) and CD4^+^CD25^+^CD127^low^/CD4^+^T (12.77 ± 5.56%) were significantly higher than those from normal controls (1.13 ± 0.16%, 9.51 ± 1.79%, 9.62 ± 5.77%, respectively, all P < 0.05). The ratio of CD4^+^/CD8^+^(r = 0.685) and CD4^+^CD25^+^/CD3^+^T(r = 0.568) were positively correlated with the quantity of osteoblasts (both P < 0.05) (Figure 3a-b).

The serum IL-7 level of MM patients (2.07 ± 0.71 ng/L) was higher than that of normal controls (1.62 ± 0.15 ng/L, P < 0.05), and was negatively correlated with the quantity of osteoblasts (r = -0.682, P < 0.01) and CD4^+^/CD8^+^ (r = -0.511, P < 0.05) (Figure 3c).

### After co-cultured with bortezomib, the numbers and function of osteoblasts from MM patients could partly recover

**Figure 4:**
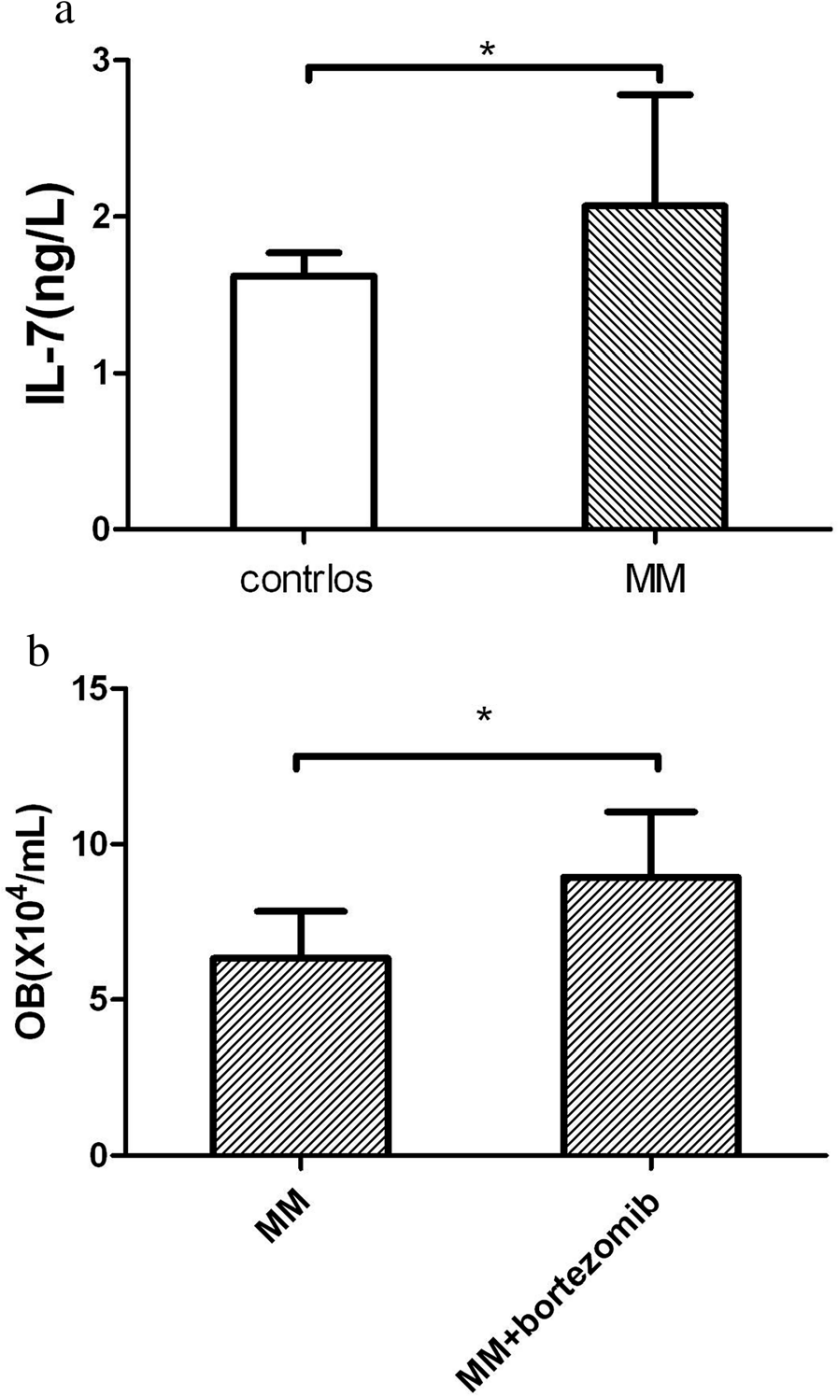
**Changes of the osteoblasts after cultured with bortezomib. (a)** After co-cultured with bortezomib, the quantity of osteoblasts from MM patients was more than those without bortezomib (*P < 0.05). **(b)** The expressions of BMP2 mRNA were detected by RT-PCR. The BMP2 expression of osteoblasts from MM patient (line a,c) was negative. The BMP2 expression of osteoblasts from normal controls (line b) and that of osteoblasts from MM patient co-cutured with Bortezomib (line d), were both positive.

After co-cultured with bortezomib, the quantity of osteoblasts from MM patients (8.94 ± 2.09) was more than that of osteoblasts from MM patients without bortezomib (6.33 ± 1.51, P < 0.05) (Figure 4a). And the calcium depositions in MM osteoblasts with bortezomib (8.83 ± 1.94) was more than those without bortezomib (P < 0.01), but less than those of normal controls (P < 0.01) (Figure 2c).The expressions of BMP-2 mRNA (308 bp) were positive in normal osteoblasts and MM patients’ osteoblasts co-cultured with bortezomib, but were negative in those of MM patients’ osteoblasts without bortezomib (Figure 4b).

## Discussion

MBD, including osteoporosis or multiple osteolytic lesions, bone pain and fractures, is the main complication of MM which due to the increased number and activativity of osteoclasts. In recent years, the molecular biology studies of MBD advanced a lot. The function and pathological changes of osteoblasts gradually become the focus of research. In BM, immune cells are derived from the hematopoietic stem cells that interact with bone cells [4]. The skeletal and immune systems are closely related through cellular and molecular interactions. The MM patients always have immunologic deficiency, and investigating their altered immunity is helpful to find the method of immunotherapy to treat MBD.

*In vitro* culture of osteoblasts has been developed for 40 years. It was shown that dexamethasone, β- glycerophosphate and VitC could induce the differentiation of osteoblasts from bone marrow mesenchymal stem cells (BMMSC) and their osteogenic function *in vitro*[5]. In this study, we used BM cells culture to induce the differentiation osteoblasts by nutrient solution with conditional factors *in vitro*. The results showed that the numbers, osteogenic potential and activativity of osteoblasts in MM patients were significant decreased.

In past studies, the Wingless-type (Wnt) signaling, osteoclasts activating factors, transcription factor Runx2 [6], receptor activator of nuclear factor-κB (RANK)/RANK ligand (RANKL)/osteoprotegerin(OPG) system, IL-3, IL-7, and tumor necrosis factor (TNF)-α all involved in the inhibition of osteoblasts in MBD. Osteoblasts produce OPG and RANKL. RANKL binds to RANK and enhances the differentiation/activativity of osteoclasts. Dickkopf-1(DKK1), a soluble inhibitor of Wnt signaling, regulates Wnt signaling by binding to the Wnt coreceptor lipoprotein-related protein-5 (LRP5). Forced overexpression of DKK1 in osteoblasts leads to osteopenia and inhibits fracture repair [7,8]. Some cytokines such as IL-7, IL-3, IL-6, TNF-α produced by myeloma cells can inhibit the differentiation and activativity of osteoblasts and induce osteoblasts apoptosis. IL-6 which produced by both BMMSC and myeloma cells is a key cytokine in the pathogenesis and disease progression of MM [9]. *In vitro*, IL-7 could inhibit the culture of CFU-E and CFU- osteoblasts and decrease the activity of RUNX2/Cbfal of preosteoblast, and the antibody against IL-7 could block the inhibition of the osteogenesis of MM [1]. MM is a plasma cell malignancy with the alted cellular immunity mediated by T cells. Previous studies have shown that DCs from MM patients significantly lower expression of HLA-DR, CD40, and CD80 antigens and impaired induction of allogeneic T-cells proliferation compared with controls [10]. It has demonstrated that DCs in MM patients have distinct function to sensitize incipient T cells, and could induce cytotoxic T cells, influence the balance between Th1 and Th2, which affect immune response to tumor. Tregs is considerable in negating accommodation of immune response and autoimmunity tolerance. Studies showed that Tregs can inhibit the immune response of tumor cells then lead to tumor growth indirectly. CD4^+^CD25^+^ Tregs are an important group of negative immunoregulation which induce the immune suppression and immune escape of tumor [11]. CD8^+^CD25^+^T cells have affinis phenotype, function and regulatory mechanism to CD4^+^CD25^+^ Tregs. It can inhibit the generation of other cell subsets [12]. Some studies showed that the cellular immunity changes were maybe one of the most important reasons for immunity impairment in MM patients.

The bone and immune system are closely related through cellular and molecular interactions [13]. RANKL is an essential cytokine for osteoclastogenesis and osteoclasts activation, and expressed by osteoblasts and activated T cells. IL-7 and IL-3 have been reported to block osteoblast differentiation. IL-7 is also a potent T cell activating cytokine that causes proliferation, survival and differentiation of T cells in the periphery to maintain homeostatic T cell balance. Study showed it induces CD4 T cell activation [14]. IL -3 acts as a bi-functional mediator of MBD, increasing osteoclasts and simultaneously suppressing osteoblast formation. IL-3 can blunt the growth of osteoblasts mediated by CD45+/CD11b + monocytes/macrophages [3]. Some medicine can affect both of these two systems. Zoledronic Acid (ZA) can inhibit activation and function of osteoclasts, interfere reabsorption of bone by osteoclasts. In recent studies, ZA-treated MM DC were highly effective in activating autologous γδ T cells, even in patients refractory to stimulation with ZA-treated monocytes [15]. All of them indicated that there exist many associations between the bone formation and immune system.

In our study, we showed that the ratio of CD4^+^/CD8^+^, DC1/DC2, Th1/Th2, and CD8^+^CD25^+^/CD3^+^T were significantly decreased, meanwhile that of CD4^+^CD25^+^/CD3^+^T and CD4^+^CD25^+^CD127^low^/CD4^+^T were significantly increased. The ratio of CD4^+^/CD8^+^ and CD4^+^CD25^+^/CD3^+^T had positive correlation with the quantity of osteoblasts. It indicated that MM patients had abnormal cellular immunity which correlated with the abnormity of osteoblasts. After co-cultured with MM patient serum, the quantity of normal osteoblasts was decreased which indicated some cytokines could inhibit the differentiation of osteoblasts. In further, we found that the serum IL-7 level of MM patients was higher than that of normal controls. And the IL-7 level was correlated with not only the quantity of osteoblasts but also the ratio of CD4^+^/CD8^+^.

Bortezomib, a clinically available proteasome inhibitor which can not only destroy MM cells but also increases osteoblast differentiation in human mesenchymal cells without affecting the number of osteoblast progenitors and the viability of mature osteoblasts. And promotes the osteoclasts formation and bone resorption. Bortezomib induce bone formation through increasing bone morphogenetic protein–2(BMP-2) produced by osteoblasts, which can increase Runx-2 levels, induce MSCs to differentiate into osteoblasts and enhance bone regeneration, and BMP-2 induces canonical Wnt signaling through autocrine activation of Wnt3a mRNA. Bortezomib can also decrease RANKL and DKK1 levels in the serum of myeloma patients. *In vivo* and *in vitro* observations support that both direct and indirect effects on bone formation process could occur during bortezomib treatment [16-18].

After co-cultured with bortezomib, we found there was no effect to osteoblasts from normal controls, but the quantity and activity of osteoblasts from MM could increase. We also found that the expression of BMP-2 mRNA was increased in MM patients’ osteoblasts after co-cultured with bortezomib which from negative to positive. It indicated that bortezomib could partly recover the proliferation and osteogenic potential of osteoblasts in MM.

In conclusion, the proliferation and osteogenic potential of osteoblasts from MM patients were decreased *in vitro* in this study. Bortezomib could increase the number and function of osteoblasts in MM. The quantity of T cells subgroup, DC, Th, and effector T cells all reduced while Tregs relatively raised which had correlation with osteoblasts in MM. And cellular immunity which secreted IL-7 in MM patient could decrease the proliferation and osteogenic potential of osteoblasts. These results indicated that there were some relationship between abnormal osteoblasts and ACI in MBD.

## Competing interests

The authors declare that they have no competing interests.

## Authors’ contributions

SG and FP carried out the cell curture, ELISA and the statistical analyses and drafted the manuscript. JL and HL carried out the Flow cytometry and PCR. HW and LX participated in the design of the study and performed the statistical analysis. RF and ZS conceived of the study, and participated in its design and coordination and helped to draft the manuscript. All authors read and approved the final manuscript.
